# Hypothermia Augments Neuroprotective Activity of Mesenchymal Stem Cells for Neonatal Hypoxic-Ischemic Encephalopathy

**DOI:** 10.1371/journal.pone.0120893

**Published:** 2015-03-27

**Authors:** Won Soon Park, Se In Sung, So Yoon Ahn, Hye Soo Yoo, Dong Kyung Sung, Geun Ho Im, Soo Jin Choi, Yun Sil Chang

**Affiliations:** 1 Department of Pediatrics, Samsung Medical Center, Sungkyunkwan University School of Medicine, Seoul, Korea; 2 Samsung Biomedical Research Institute, Sungkyunkwan University School of Medicine, Seoul, Korea; 3 Biomedical Research Institute, MEDIPOST Co., Ltd., Seoul, Korea; Hôpital Robert Debré, FRANCE

## Abstract

Though hypothermia is the only clinically available treatment for neonatal hypoxic-ischemic encephalopathy (HIE), it is not completely effective in severe cases. We hypothesized that combined treatment with hypothermia and transplantation of human umbilical cord blood (UCB)-derived mesenchymal stem cells (MSCs) would synergistically attenuate severe HIE compared to stand-alone therapy. To induce hypoxia-ischemia (HI), male Sprague-Dawley rats were subjected to 8% oxygen for 120 min after unilateral carotid artery ligation on postnatal day (P) 7. After confirmation of severe HIE involving >50% of the ipsilateral hemisphere volume as determined by diffusion-weighted brain magnetic resonance imaging (MRI) within 2 h after HI, intraventricular MSC transplantation (1 × 105 cells) and/or hypothermia with target temperature at 32°C for 24 h were administered 6 h after induction of HI. Follow-up brain MRI at P12 and P42, sensorimotor function tests at P40–42, evaluation of cytokines in the cerebrospinal fluid (CSF) at P42, and histologic analysis of peri-infarct tissues at P42 were performed. Severe HI resulted in progressively increased brain infarction over time as assessed by serial MRI, increased number of cells positive for terminal deoxynucleotidyl transferase nick-end labeling, microgliosis and astrocytosis, increased CSF cytokine levels, and impaired function in behavioral tests such as rotarod and cylinder tests. All of the abnormalities observed in severe HIE showed greater improvement after combined treatment with hypothermia and MSC transplantation than with either therapy alone. Overall, these findings suggest that combined treatment with hypothermia and human UCB-derived MSC transplantation might be a novel therapeutic modality to improve the prognosis of severe HIE, an intractable disease that currently has no effective treatment.

## Introduction

Despite recent improvements in perinatal and neonatal intensive care medicine, birth asphyxia and the ensuing hypoxic ischemic encephalopathy (HIE) remain a major cause of neonatal mortality or permanent neurologic morbidity, including cerebral palsy, mental retardation, learning disabilities, and epilepsy, in survivors[1, 2]. Currently, hypothermia is the only clinically effective intervention that improves the prognosis of neonatal HIE[3–5]. However, even with hypothermia treatment, approximately 50% of newborn infants with HIE die or suffer from significant neurological disabilities. Outcomes are even worse for the severe type of neonatal HIE[6, 7]. Therefore, additional treatments other than hypothermia that maximize neuroprotection and improve the prognosis of severe neonatal HIE are urgently needed.

Recently, several studies reported the neuroprotective effects of exogenously administered mesenchymal stem cells (MSCs) in a newborn animal model of HIE[8–10]. Among various sources of MSCs, including bone marrow and adipose tissue, umbilical cord blood (UCB) is most promising because of its availability, lack of ethical concerns, high proliferation capacity[11, 12], and low immunogenicity[13]. We have shown that intraventricular transplantation of human UCB-derived MSCs significantly attenuates even severe brain injury involving >50% of the ipsilateral hemisphere induced by middle cerebral artery occlusion in rat pups[14]. Because of the similarity between the pathophysiology of neonatal HIE and that of stroke, the favorable experimental results of MSC transplantation observed in neonatal stroke might be extrapolated for future translation to the treatment of neonatal patients with severe HIE. Kaneko et al. reported that combined treatment with moderate hypothermia and MSCs significantly improved cell survival and mitochondrial activity after *in vitro* oxygen glucose deprivation[20]. Taken together, these findings suggest that combined treatment with hypothermia and human UCB-derived MSC transplantation might induce superior neuroprotective effects compared with either stand-alone therapy, especially for the treatment of severe neonatal HIE. However, little is known about the outcomes of combined therapy with MSC transplantation and hypothermia for severe HIE, and to date, no *in vivo* study has been performed to investigate this therapy.

Therefore, in this study, we compared the therapeutic efficacy of combined hypothermia and human UCB-derived MSC transplantation with that of either stand-alone hypothermia or MSC transplantation for severe HIE in newborn rats. After confirming severe HIE brain injury involving >50% of the ipsilateral hemisphere using diffusion-weighted (DW) brain magnetic resonance imaging (MRI), rat pups were randomly allocated among experimental groups. The therapeutic efficacy of each treatment was evaluated using serial brain MRI monitoring *in vivo*, and primary endpoint is improvement of the volume ratio of intact ipsilateral hemisphere to contralateral hemisphere. Secondary endpoint were attenuation of cell death (TUNEL), reactive gliosis (GFAP), and inflammatory cytokines.

## Materials and Methods

This study was approved by the Institutional Review Board of Samsung Medical Center and by Medipost Co., Ltd., Seoul, Korea. All experimental protocols were approved by the Institutional Animal Care and Use Committee of Samsung Biomedical Research Institute, and the study followed institutional and National Institutes of Health guidelines for laboratory animal care.

### Cell Preparation

Human UCB was obtained from healthy normal full-term newborns after obtaining written informed parental consent, and MSCs were isolated and expanded according to a previously reported procedure[11, 15]. Fifth-passage human UCB-derived MSCs from a single donor were transplanted. MSCs were co-cultured with *micron-sized paramagnetic iron-oxide (MPIO)* particles (Bangs Laboratories, Fishers, IN, USA) in culture medium in order to induce endocytosis before transplantation according to the manufacturer’s protocol, and localization of transplanted donor cells was confirmed as described in the Supporting Information.

### Animal Model and ethics statement

**Fig 1:**
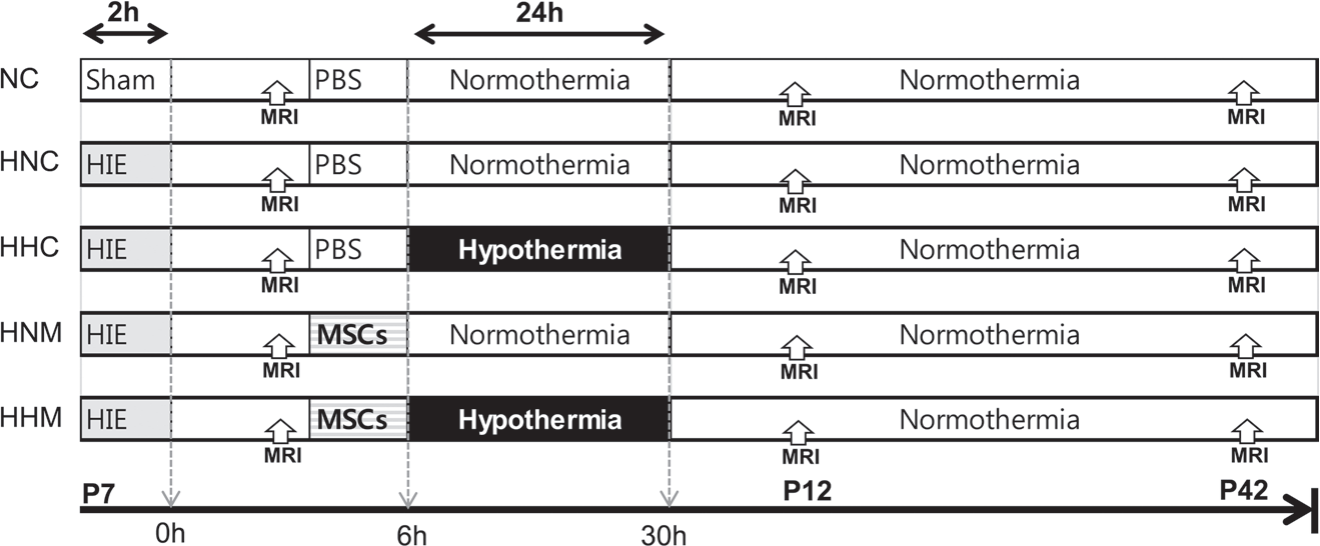
Experimental protocol.

All animal procedures were performed in an AAALAC-accredited specific pathogen-free facility. Fig. 1 shows details of the experimental schedule. Sprague-Dawley male rats (Orient Co., Seoul, Korea) at postnatal day (P) 7 were used and raised by dam rats in the standard cage, 50 liter Plexiglas chamber except the hypothermia period. Dam rats could assess to water and laboratory chow freely, and were maintained in an alternating 12-hour light/dark cycle with constant room humidity and temperature. We assessed and monitored the condition of rat pups on a weekly basis regularly and four times per day in a daily basis especially for the seven days after modeling. Humane endpoint is described in the Supporting Information. Cerebral hypoxia-ischemia (HI) was induced by unilateral carotid artery ligation and exposure to 8% oxygen for 120 min. After confirmation of severe brain injury involving more than 50% of the ipsilateral hemisphere volume as determined by DWI brain MRI performed within 2 h after HI, rat pups were randomly allocated among four experimental groups as follows: HI with normothermia control group (HNC, n = 11); HI with hypothermia control group (HHC, n = 10); HI with normothermia MSC transplantation group (HNM, n = 12); and HI with hypothermia MSC transplantation group (HHM, n = 10). A normoxia normothermia control group underwent a sham operation (NNC, n = 5). MSCs (1 × 10^5^ cells in 10 l saline) were injected into the ipsilateral ventricel to injury, and/or hypothermia with a target temperature of 32°C for 24 h [16] was initiated 6 h after HI. The dose of the MSCs was referred to our previous study of MSCs transplantation in the newborn rat model of perinatal brain injury [14].

Follow-up brain MRI was performed at P12 and P42, and behavioral function tests were performed at P40–42. At P42, cerebrospinal fluid (CSF) was obtained by cisternal tap for enzyme-linked immunosorbent assay (ELISA) of cytokines, the rats were sacrificed under the deep pentobarbital (Entobar, Hanlim Pharmaceutical C7-8o., Seoul, Korea) anesthesia (60 mg/kg, intraperitoneal), and brain tissue was obtained for histologic analyses. Details of the induction of HI, MRI, hypothermia, MSC transplantation, behavioral functional tests, brain histologic analysis, and ELISA protocols are provided in the Supporting Information.

### Statistical Analysis

Data are expressed as mean ± standard error of the mean (SEM). Survival rates were compared by Kaplan-Meier analysis followed by a log-rank test. For continuous variables, statistical comparison between groups was performed by one-way analysis of variance (ANOVA) and Tukey’s post hoc analysis. For time-course variables, repeated-measures ANOVA with Tukey's post hoc comparison was used. A *P* value < 0.05 was considered significant. All data were analyzed using SPSS version 17.0 (IBM, Chicago, IL, USA).

## Result

### Survival Rates and Body Weights

Survival rates were not significantly different among HIE groups: NNC, 5/5 (100%); HNC, 8/11 (73%); HHC, 7/10 (70%); HNM, 9/12 (75%); and HHM 8/10 (80%). The final cause of death was mainly originated from the inappropriate sucking ability and poor oral feeding related with the severe brain infarction, and no rat pups met the humane endpoints before the end of the present study. Growth retardation observed in the HNC compared to the NNC group was significantly attenuated in both the HNM and HHM groups but not in the HHC group (S2 Fig).

### Serial Brain MRI and Injury Assessment

**Fig 2:**
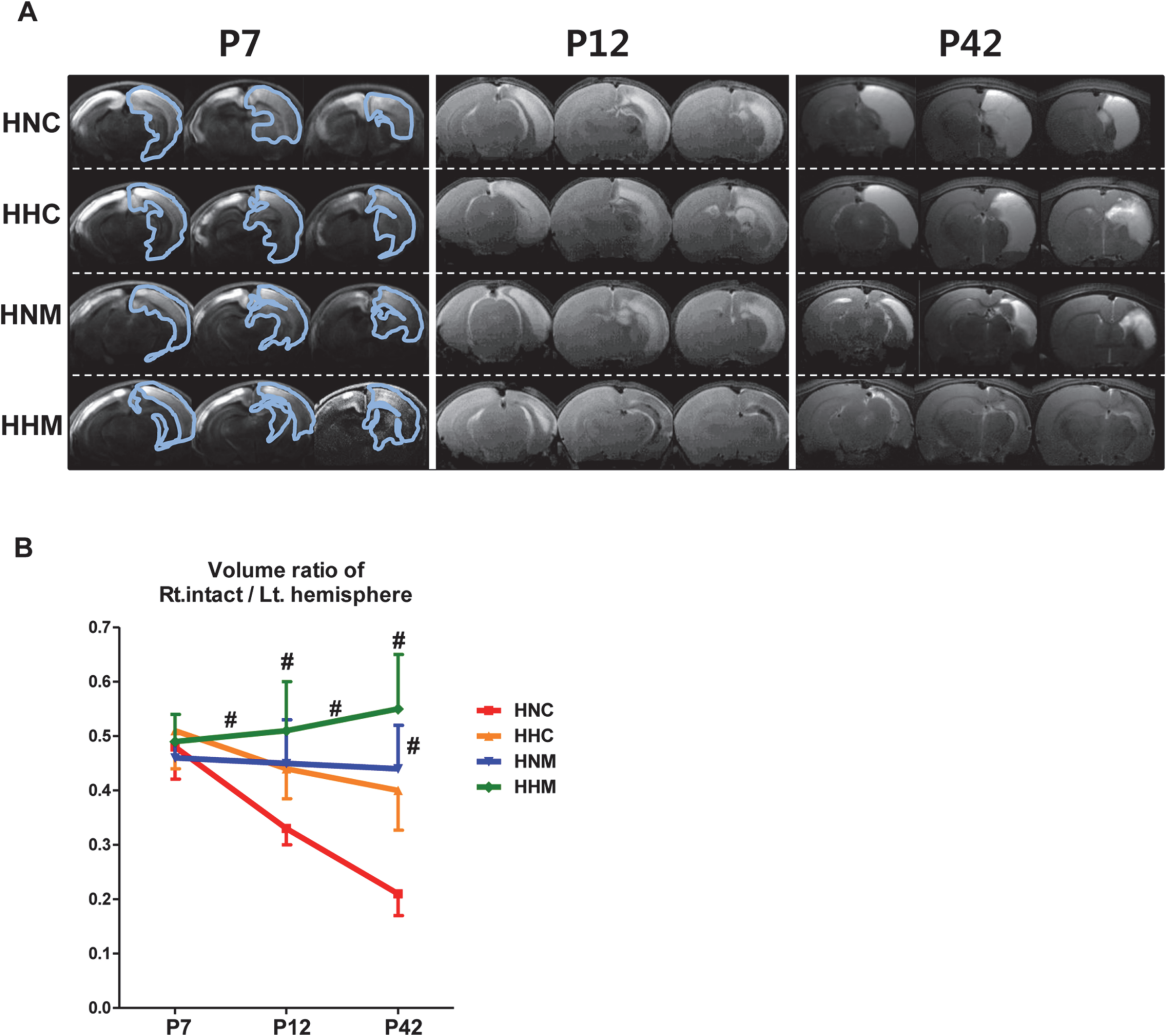
Combined hypothermia and mesenchymal stem cell transplantation attenuated the progression of ischemic brain lesions after severe HIE. (A) Representative serial brain MRI images from treatment groups at P7, P12, and P42 (0, 5, and 35 days after inducing HIE, respectively). (B) Volume ratio of the ipsilateral intact area to the contralateral contralateral hemisphere area measured by MRI. Data are mean ± SEM. HNC, HIE+normothermia control group (n = 8); HHC, HIE+hypothermia group (n = 7); HNM, HIE+normothermia+MSCs group (n = 9); HHM, HIE+hypothermia+MSCs group (n = 8). # P < 0.05 vs. HNC.

Representative serial *in vivo* brain MRI findings performed at P7, P12, and P42 (2 hours, 5 days, and 35 days after HI, respectively) for animals in each experimental group are shown in Fig. 2A. Although the initial ipsilateral intact brain volume measured at P7 was not significantly different among the study groups, the intact brain volume in the HNC group progressively decreased over time on follow-up brain MRI at P12 and P42 (Fig. 2B). The reduction in intact brain volume observed in HNC rats was significantly attenuated in the HNM and HHM groups but not in HHC (P42; 0.21±0.12, 0.41±0.14, 0.44±0.30, and 0.55±0.27 in HNC, HHC, HNM, and HHM respectively; HNC vs. HNM, *P*<0.05; HNC vs. HHM; *P*<0.01), with better attenuation in the HHM group than in the HNM group (changes in ipsilateral intact brain volume from P7 to P12 and from P12 to P42; HNC vs HNM, *P>*0.05; HNC vs HHM, *P*<0.05).

### Confirmation of Donor Cells

T2*-weighted imaging at P42 revealed areas of low signal intensity, indicating the presence of transplanted MPIO-tagged MSCs in the peri-infarct area in both the HNM and HHM groups but not in the HNC or HHC group (S3 Fig). Signal along the ischemic boundary area at P42 (35 days after inducing severe HIE) suggests that MSCs migrated to the injured area from the right ventricle. Double merged cells with both of MPIO green fluoroscence and NeuN or GFAP in the brain coronal sections were not observed and no abnormal pathologic feature like mass lesion or tumor-like agllomerates was found in the brain histology sections.

### Cell Death and Reactive Gliosis

**Fig 3:**
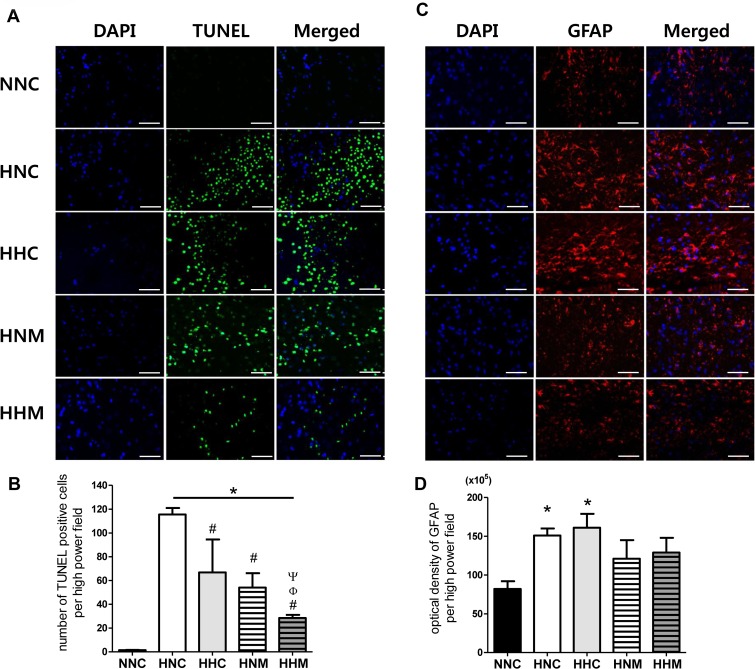
Combined hypothermia and mesenchymal stem cell transplantation ameliorated the increase in cell death and reactive gliosis in the brain after severe HIE. The images show representative immunofluorescence micrographs of the penumbra area with staining for TUNEL (A), glial fibrillary acidic protein (GFAP) (C), and DAPI (original magnification; ×400, scale bars; 25 μm). Data are average number of TUNEL-positive cells (B) and average density of GFAP staining (D) in the penumbra area, presented as mean ± SEM. NNC, sham control group (n = 5); HNC, HIE+normothermia control group (n = 8); HHC, HIE+hypothermia group (n = 7); HNM, HIE+normothermia+MSCs group (n = 9); HHM, HIE+hypothermia+MSCs group (n = 8). * P < 0.05 vs. NNC, # P < 0.05 vs. HNC, Φ P < 0.05 vs. HHC, Ψ P < 0.05 vs. HNM.

The marked increase in TUNEL-positive cells observed in the HNC group compared to the NNC group was significantly improved in the HHC, HNM, and HHM groups, with better attenuation in the HHM group than in the HHC or HNM groups (Fig. 3A&B).

The increase in GFAP level, indicative of reactive gliosis, observed in the HNC group compared to the NNC group was improved in both the HNM and HHM groups but not in the HHC group (Fig. 3C&D).

### Inflammation

**Fig 4:**
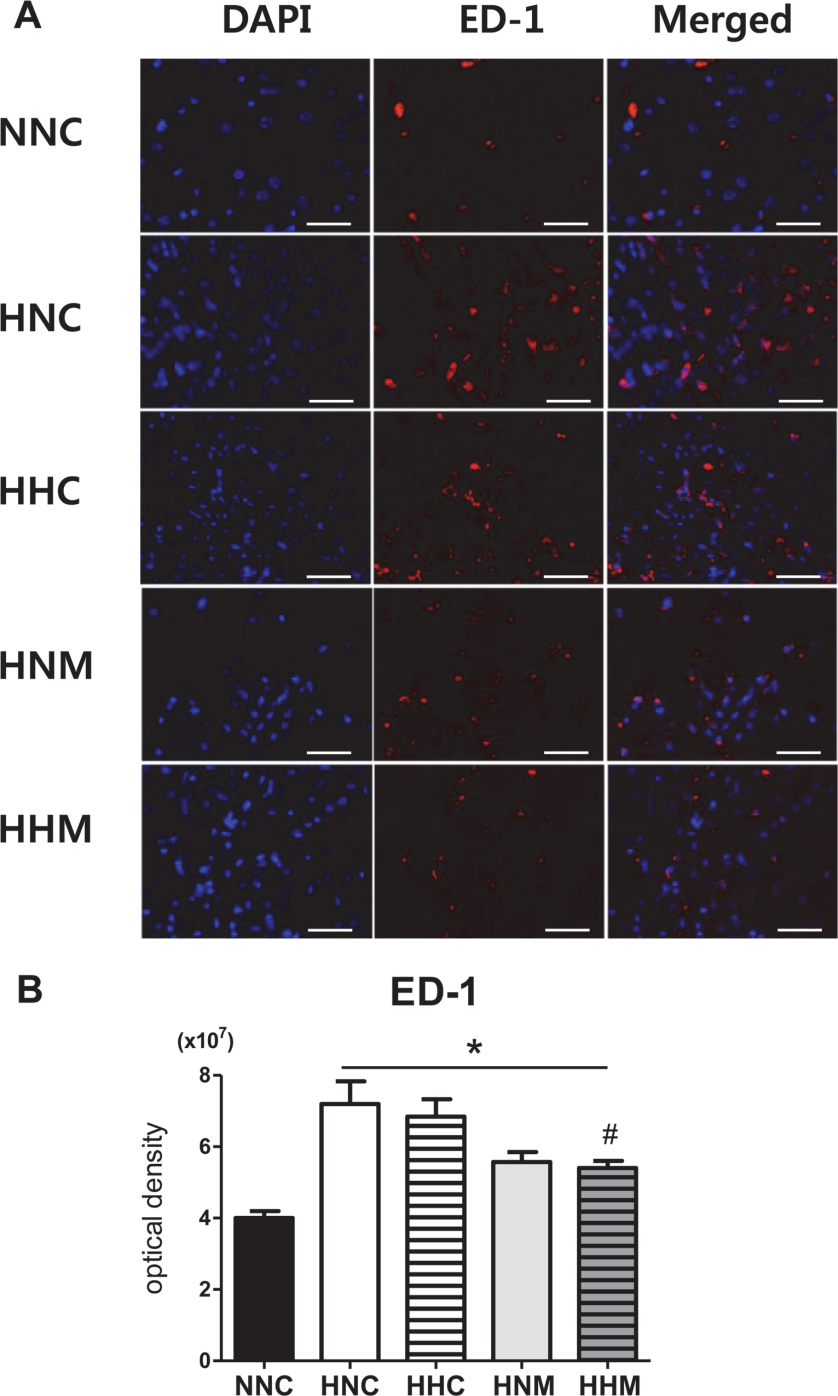
Combined hypothermia and mesenchymal stem cell transplantation reduced brain inflammation as represented by active macrophages after severe HIE. (A) Representative immunofluorescence micrographs of the penumbra area with staining for ED-1 (red) and DAPI (blue) (original magnification; ×400, scale bars; 25 μm). Average optical density of ED-1 staining (B) in the penumbra area. Data are mean ± SEM. NNC, sham control group (n = 5); HNC, HIE+normothermia control group (n = 8); HHC, HIE+hypothermia group (n = 7); HNM, HIE+normothermia+MSCs group (n = 9); HHM, HIE+hypothermia+MSCs group (n = 8). *P < 0.05 vs. NNC, # P < 0.05 vs. HNC.

Increased optical density of ED-1-positive cells which indicated activated microglia in the peri-infarct area observed in the HNC group compared to the NNC group was significantly improved in the HHM group but not in the HHC or HNM group (Fig. 4).

**Fig 5:**
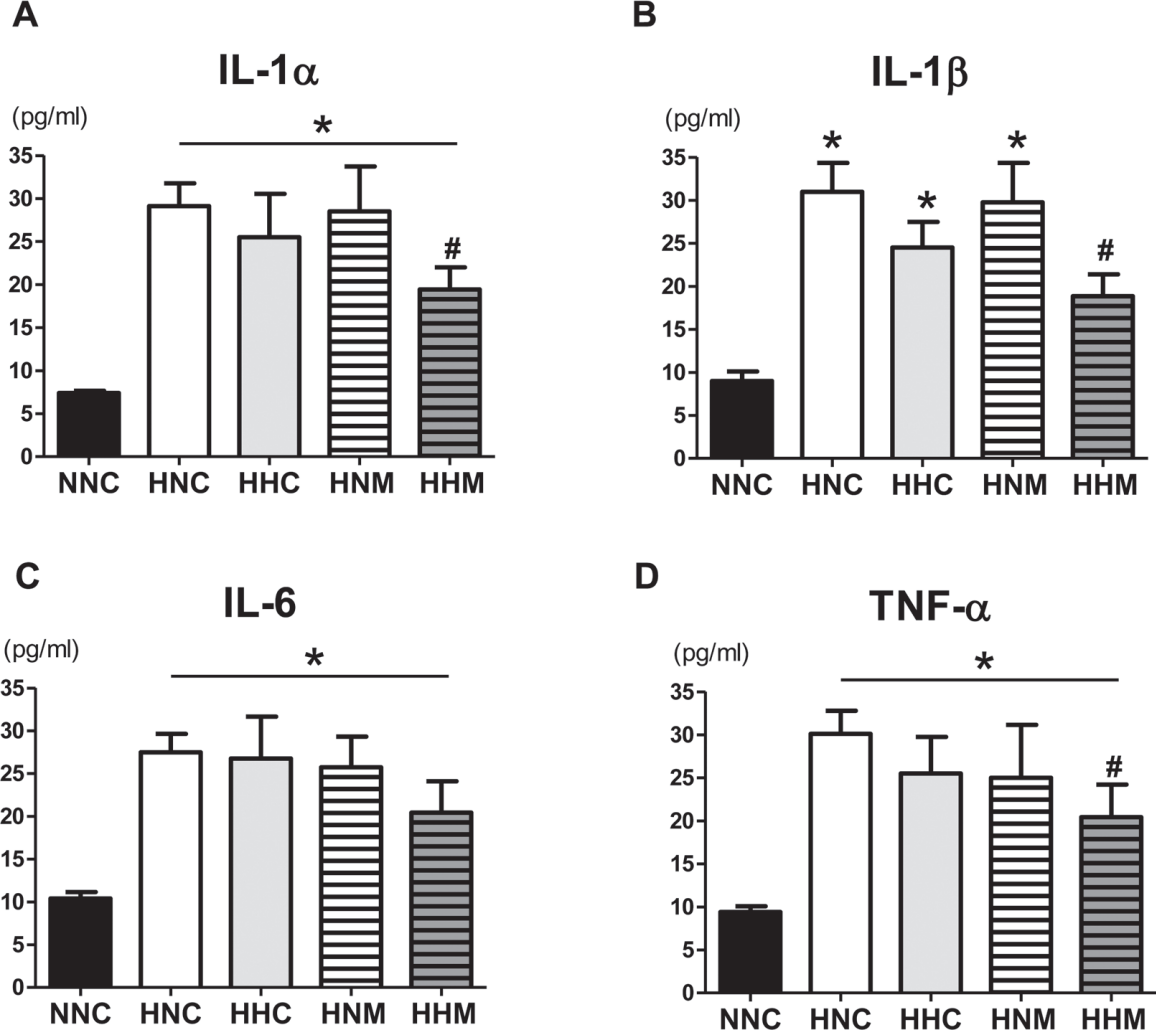
Concentrations of inflammatory cytokines IL-1α, IL-β, IL-6, and TNF- α in cerebrospinal fluid at P42. Data are mean ± SEM. NNC, sham control group (n = 5); HNC, HIE+normothermia control group (n = 8); HHC, HIE+hypothermia group (n = 7); HNM, HIE+normothermia+MSCs group (n = 9); HHM, HIE+hypothermia+MSCs group (n = 8). *P < 0.05 vs. NNC, # P < 0.05 vs. HNC.

Similarly, the significant increases in concentrations of cytokines such as IL-1α, IL-1β, IL-6, and TNF-α in the CSF of animals in the HNC group compared to the NNC group at P42 were significantly improved in the HHM group but not in the HHC or HNM group (Fig. 5).

### Functional Behavior Tests

**Fig 6:**
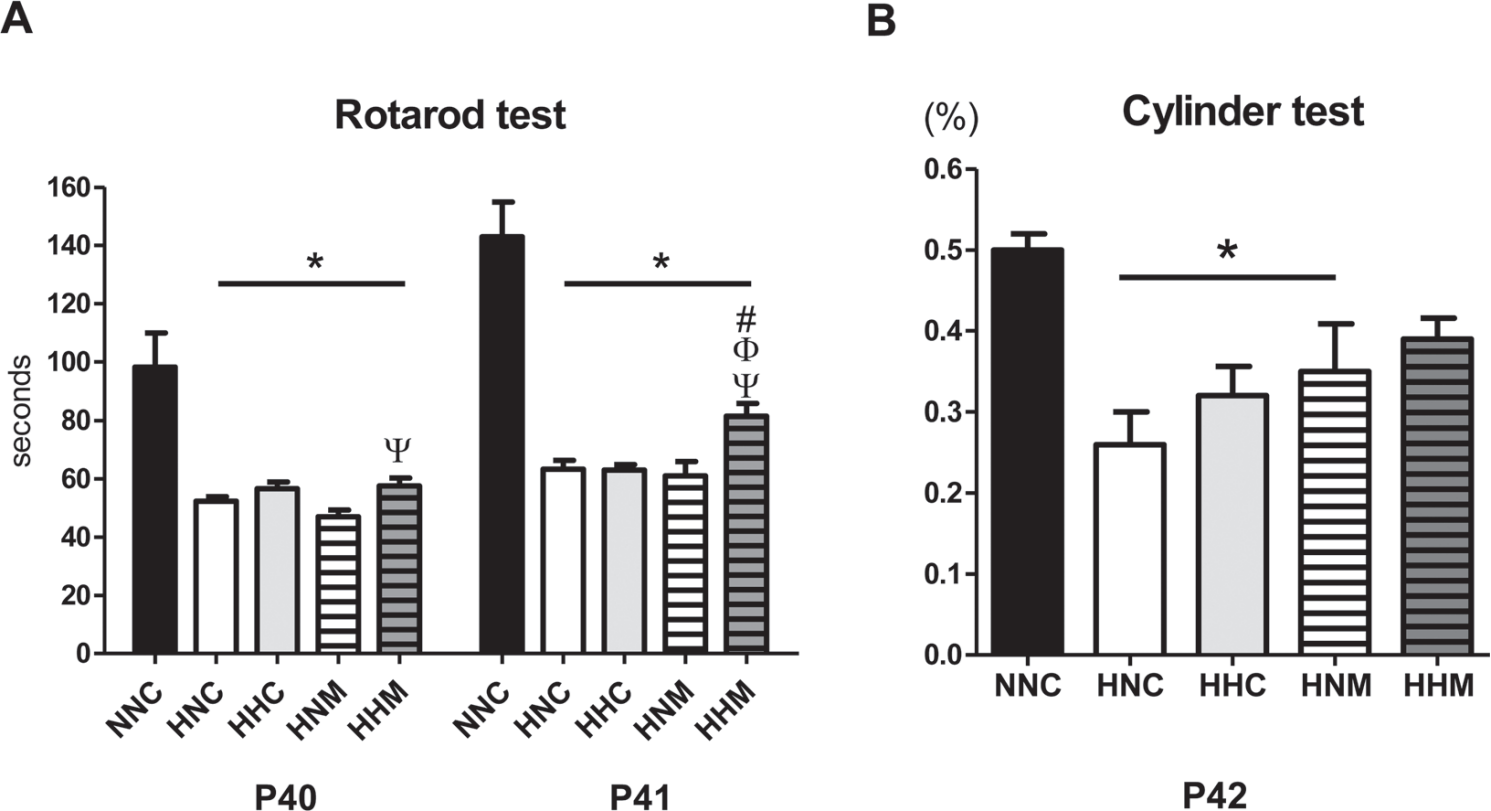
Combined hypothermia and mesenchymal stem cells transplantation improved impaired sensorimotor function after severe HIE. Sensorimotor functional outcomes on rotarod (A) and cylinder (B) tests. Data are mean ± SEM. NNC, sham control group (n = 5); HNC, HIE+normothermia control group (n = 8); HHC, HIE+hypothermia group (n = 7); HNM, HIE+normothermia+MSCs group (n = 9); HHM, HIE+hypothermia+MSCs group (n = 8). * P< 0.05 vs. NNC, # P < 0.05 vs. HNC, Φ < 0.05 vs. HHC, Ψ P < 0.05 vs. HNM.

To assess sensorimotor functions, a rotarod test was performed at P40 and P41, and a cylinder test was performed at P42[14, 17]. In the rotarod test at P40, a shorter latency to fall was observed in the HNC group compared to the NNC group and was slightly, yet significantly, improved in the HHM group but not in the HHC or HNM group (Fig. 6A). In a follow-up rotarod test at P41, the latency to fall was significantly improved in both NNC and HHM groups but not in the HNC, HHC, or HNM group. In the cylinder test at P42, the lower frequency of left paw use observed in the HNC group compared to the NNC group was improved only in the HNM group but not in the HHC or HNM groups (Fig. 6B).

## Discussion

Development of an appropriate animal model that simulates clinical neonatal HIE is essential in order to elucidate the pathogenesis of HIE and determine the effectiveness of therapeutic interventions[18]. In the present study, the Vannucci model of HIE in the 7-day-old rat was used to induce cerebral HI[19] because it is a well-known standardized model for evaluating the therapeutic efficacy of neuroprotective strategies such as hypothermia[16] or MSC transplantation[20] for neonatal HIE. Because of wide variability in brain injury in the model[21], the animals were randomly allocated into study groups only after confirming severe HIE involving >50% of the ipsilateral hemisphere as determined by DW brain MRI within 2 h after induction of HI. As presented before by Ashwal, et al.[22], we sometimes observed contralateral hemispheric involvement only in the DWI brain images taken 2h after HIE modeling, but not in the follow-up T2-weighted brain MRI performed at P12 and P42. We thus included only the severe HIE rats defined as brain injury involving more than 50% of the ipsilateral hemisphere regardless of the contralateral hemispheric involvement in the present study

In the present study, therapeutic hypothermia with a target temperature of 32°C for 24 h started 6 h after induction of HI (HHC) significantly attenuated the number of TUNEL-positive cells compared with the HNC rats but failed to significantly improve the severe brain injury as assessed by progressively increased brain infarct volume, astroglial reaction, and abnormal behavior tests. Hypothermia was started 6 h after induction of HI because this time period falls within the therapeutic time window[23], and a delay of up to 6 h in detecting the signs of HIE and/or applying hypothermia is common and tolerable in clinical practice. The CoolCap Trial found that the therapeutic efficacy of hypothermia was dependent on the severity of HIE, with protection achieved only in cooled newborns with moderate brain injury[24]. Sabir et al. reported no neuroprotective effect of hypothermia, even when it was started immediately after severe neonatal HIE in newborn rats[25]. Overall, these findings suggest that the severity of neonatal HIE rather than the timing or duration of hypothermia is the critical determinant of the therapeutic efficacy of hypothermia, and thus additional treatments other than therapeutic hypothermia are urgently needed to maximize neuroprotection and improve the outcome of intractable severe neonatal HIE. Our animal model of sever HIE with no significant improvement with hypothermia treatment seems to simulate the clinical condition showing no significant neuroprotection of severe neonatal HIE patients with hypothermia treatment.

The major findings of this study are described below. Although MSC transplantation alone (HNM) significantly attenuated the progressive increase in brain infarct volume measured by serial brain MRI and the increased number of TUNEL-positive cells in the penumbra observed in the HNC group, combination treatment with hypothermia augmented the neuroprotective effects of MSC transplantation. Moreover, the optical density of ED-1 positive cells, the level of inflammatory cytokines, and the results of behavioral tests such as rotarod test and cylinder test were significantly improved only with combined treatment of hypothermia and MSC transplantation (HHM). To our knowledge, this is the first *in vivo* study confirming the synergistic therapeutic effects of combined treatment with hypothermia and human UCB-derived MSC transplantation.

We previously showed that apoptosis is critical for the development of brain infarction, and that inhibition of apoptosis significantly reduces late development of brain infarctions in a newborn rat model of cerebral HI[26]. In the present study, an increased number of TUNEL-positive cells was observed in rats with severe HIE and was significantly attenuated by hypothermia, MSC transplantation, and combined hypothermia plus MSC transplantation, with the greatest attenuation in the combined treatment group. These findings support the hypothesis that either hypothermia[3] or MSC transplantation[17] alone has anti-apoptotic effects, but combination therapy has greater anti-apoptotic and anti-infarction effects that might be primarily responsible for a better therapeutic outcome.

Although an elevated GFAP level indicative of increased astrocytic gliosis is a specific biomarker for neonatal HIE severity, such a level is detected only after brain injury has occurred[27]. In this study, increased GFAP level was observed in only the HHC group and not in HNM and HHM rats. Our data support the hypothesis that combined treatment with hypothermia and human UCB-derived MSC transplantation is superior to hypothermia alone in protecting against severe neonatal HIE.

After neonatal HIE, hypoxic ischemic insult might initiate an inflammatory response without concurrent infection, leading to brain injury[28]. In the present study, an increase in brain microglia positive for ED-1 and increased levels of inflammatory cytokines in the CSF were observed in HNC rats. This augmented inflammatory response was significantly improved in HHM rats but not in HHC or HNM rats. These findings supported the notion that, although both hypothermia and MSC transplantation have similar anti-inflammatory effects[3, 29], stand-alone treatment of either is insufficient to block inflammation, whereas combination treatment induced superior anti-inflammatory effects against severe neonatal HIE.

In addition to improving infarct volume and histology, improving functional outcome is important for clinical translation of combined hypothermia and human UCB-derived MSC transplantation therapy for severe neonatal HIE. In this study, abnormal behavioral results on functional tests, such as a short latency to fall in a rotarod test and a lower frequency of using the left paw in a cylinder test, were observed in HNC rats. Sensorimotor function was significantly improved in HHM rats but not in HHC or HNM rats. These findings confirm that combining hypothermia with human UCB-derived MSC transplantation was therapeutically efficacious and led to better clinical outcomes than either stand-alone therapy. Moreover, the results of behavioral function tests at P40–42 implied that neuroprotection against severe neonatal HIE might persist into adolescence[30]. Despite halving of the infarct volume, single treatment with MSCs transplantation or hypothermia could not induce any improvement in behavior function. This finding may implicate that other factors, besides intact brain volume, such as improved myelination is involved for sensorimotor function improvement.

Human UCB-derived MSC transplantation alone significantly attenuated severe neonatal brain injury in this and earlier studies[14, 17]. Combining transplantation with hypothermia potentiated the therapeutic efficacy of MSC transplantation. In addition to additive anti-apoptotic and anti-inflammatory effects, the superior neuroprotective effects and better outcomes with combined treatment compared to stand-alone therapy might also be attributable to the converging delta opioid pathway and upregulation of growth factors[31]. Further studies are needed to test this hypothesis.

Although we observed superior neuroprotective effects with combined hypothermia and MSC transplantation in this study, further extensive studies are necessary, including the determination of optimal dosage, route, timing, and safety under hypothermic conditions, for successful clinical translation of these favorable experimental results into neonatal clinical practice.

In summary, combined treatment with hypothermia and human UCB-derived MSC transplantation attenuated the development of cerebral infarctions and improved behavioral function tests after severe neonatal HIE compared to either stand-alone therapy. The neuroprotective effects exerted by the combined treatment might be primarily mediated by additive anti-apoptotic and anti-inflammatory effects. Our data suggest that combined treatment with hypothermia and MSC transplantation might be a novel therapeutic modality for severe brain injury from neonatal HIE, for which effective treatments have not yet been established. Further clinical studies are warranted.

## Supporting Information

S1 Fig Rectal temperature in experimental groups. Temperatures in each group remained stable during the intervention and were significantly different between normothermia and hypothermia groups at each measurement. Data are mean ± SEM. HNC, HIE+normothermia control group; HHC, HIE+hypothermia group; HNM, HIE+normothermia+MSCs group; HHM, HIE+hypothermia+MSCs group. * P < 0.05 vs. normothermia groups (NNC, HNC, HNM).
(TIF)

S2 Fig Weekly body weight in experimental groups. Data are mean ± SEM. HNC, HIE+normothermia control group; HHC, HIE+hypothermia group; HNM, HIE+normothermia+MSCs group; HHM, HIE+hypothermia+MSCs group. * P < 0.05 vs. NNC, # P < 0.05 vs. HNC, Φ < 0.05 vs. HHC.
(TIF)

S3 Fig Localization of grafted human UCB-derived MSCs tagged with green fluorescent micron-sized paramagnetic iron-oxide (MPIO) particles along the brain penumbra area. Donor cells were confirmed by T2* MRI as low signal-intensity indicating MPIO and green fluorescence positivity in the penumbra area.
(TIF)

S1 Methods Supplementary methods. (DOCX)
