# Supplementary figures and images for: Hypothermia Augments Neuroprotective Activity of Mesenchymal Stem Cells for Neonatal Hypoxic-Ischemic Encephalopathy
Source: PLoS One. 2015 Mar 27;10(3):e0120893. doi: 10.1371/journal.pone.0120893 (PMC4376738; doi:10.1371/journal.pone.0120893)

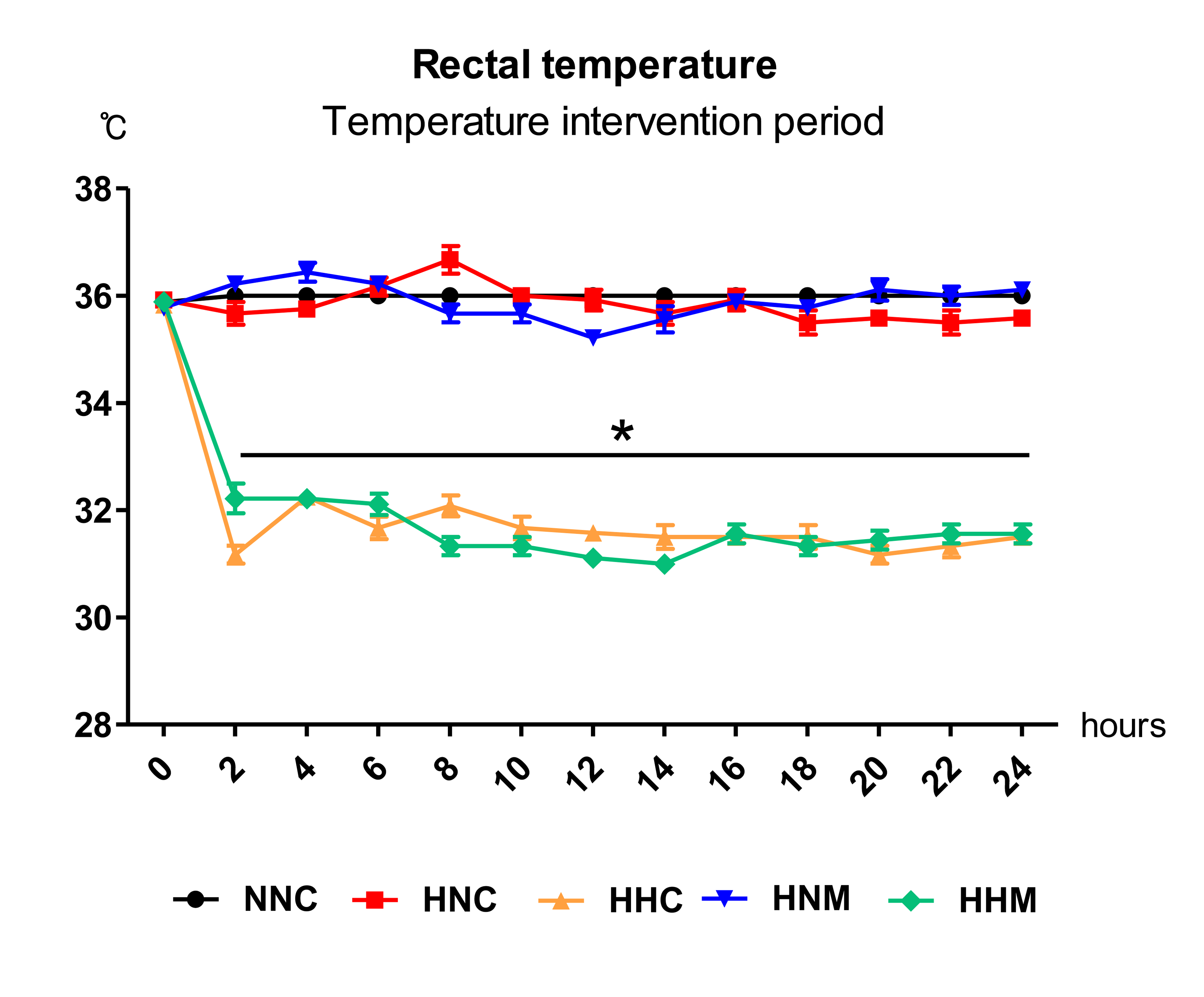

Supplement: S1 Fig — Temperatures in each group remained stable during the intervention and were significantly different between normothermia and hypothermia groups at each measurement. Data are mean ± SEM. HNC, HIE+normothermia control group; HHC, HIE+hypothermia group; HNM, HIE+normothermia+MSCs group; HHM, HIE+hypothermia+MSCs group. * P < 0.05 vs. normothermia groups (NNC, HNC, HNM). (TIF) [file pone.0120893.s001.tif]

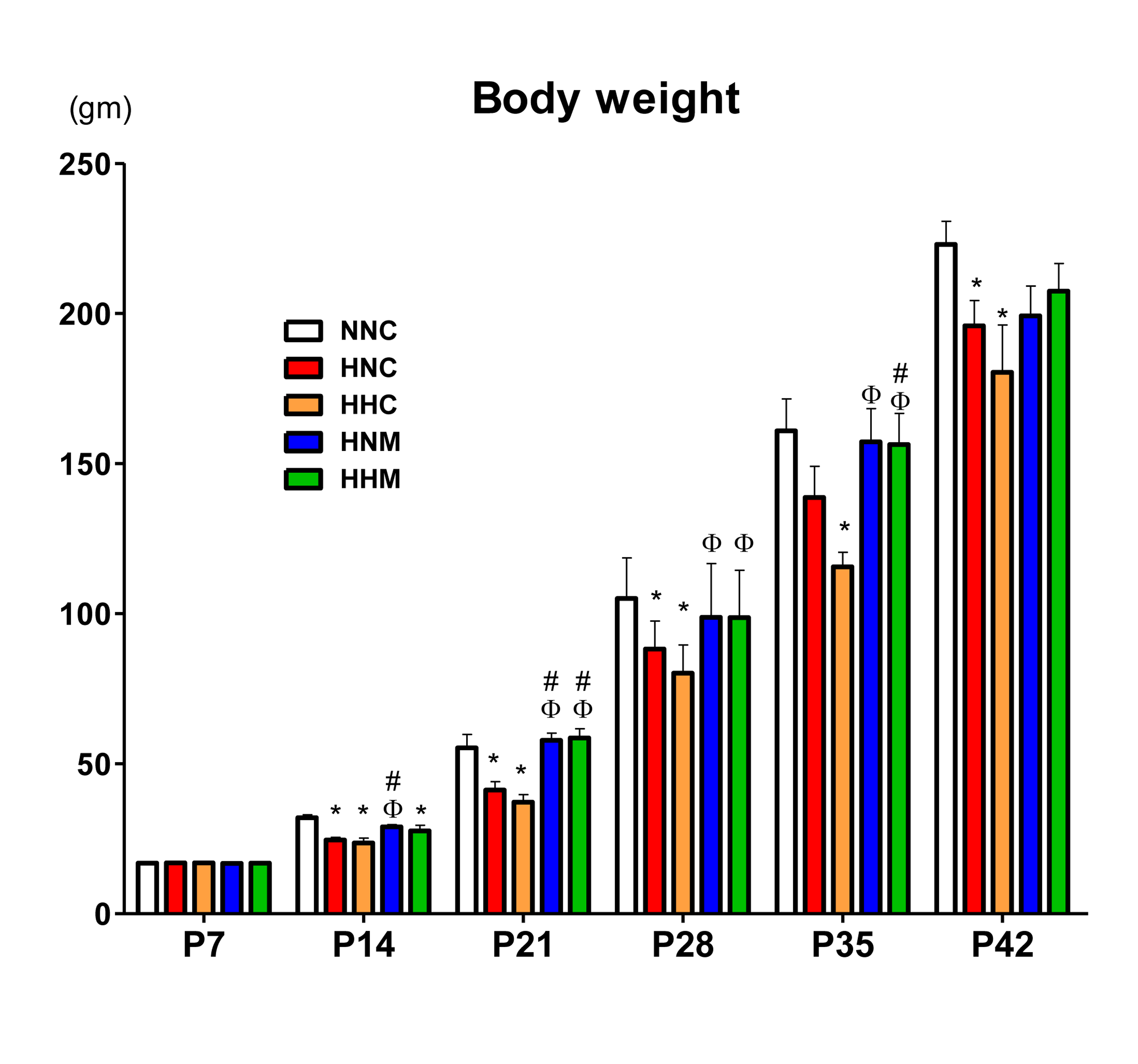

Supplement: S2 Fig — Data are mean ± SEM. HNC, HIE+normothermia control group; HHC, HIE+hypothermia group; HNM, HIE+normothermia+MSCs group; HHM, HIE+hypothermia+MSCs group. * P < 0.05 vs. NNC, # P < 0.05 vs. HNC, Φ < 0.05 vs. HHC. (TIF) [file pone.0120893.s002.tif]

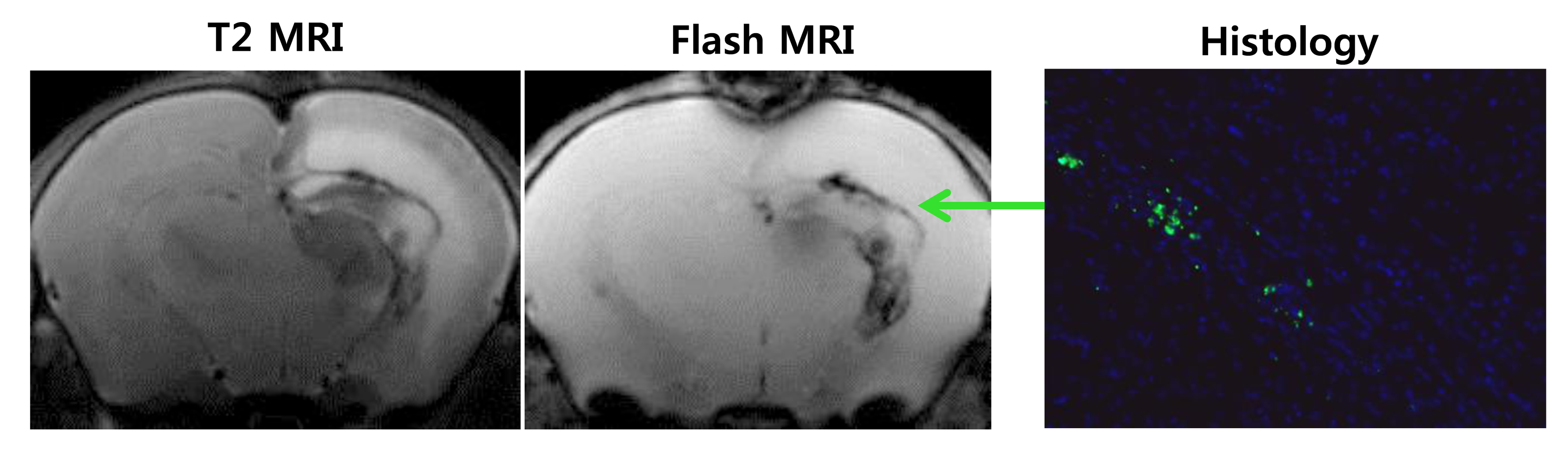

Supplement: S3 Fig — Donor cells were confirmed by T2* MRI as low signal-intensity indicating MPIO and green fluorescence positivity in the penumbra area. (TIF) [file pone.0120893.s003.tif]
