# Supplementary material for: Hypothermia Augments Neuroprotective Activity of Mesenchymal Stem Cells for Neonatal Hypoxic-Ischemic Encephalopathy
Source: PLoS One. 2015 Mar 27;10(3):e0120893. doi: 10.1371/journal.pone.0120893 (PMC4376738; doi:10.1371/journal.pone.0120893)
Supplement: S1 Methods — (DOCX) [file pone.0120893.s004.docx]

**Supporting Information**

**Hypothermia augments neuroprotective activity of mesenchymal stem cells in neonatal hypoxic-ischemic encephalopathy**

**Methods**

*Human Umbilical Cord Blood-Derived Mesenchymal Cells*

Human umbilical cord blood (UCB)-derived mesenchymal cells (MSCs) express CD105 (99.6%) and CD73 (96.3%) but not CD34 (0.1%), CD45 (0.2%), or CD14 (0.1%)[[1](#_ENREF_1)]. They are positive for human leukocyte antigen (HLA)-AB (96.8%) but not HLA-DR (0.1%). UCB-derived MSCs also express the pluripotency markers octamer-binding transcription factor 4 (Oct 4; 30.5%)[[2](#_ENREF_2)] and stage-specific embryonic antigen 4 (SSEA-4; 67.7%)[[3](#_ENREF_3)]. Human UCB-derived MSCs differentiate into the respiratory epithelium, osteoblasts, chondrocytes, and adipocytes in response to specific *in vitro* induction stimuli[[1-5](#_ENREF_1)]. We confirmed differentiation potential and karyotypic stability of human UCB-MSCs up to passage 11.

*Induction of Cerebral Hypoxia-Ischemia and Humane endpoint in animal research*

Seven-day-old male Sprague-Dawley rats received inhaled anesthesia that was a mixture of halothane and 2:1 nitrous oxide: oxygen. An incision was made in the neck along the midline, and the right common carotid artery was permanently ligated with 4–0 silk. The total time for surgery did not exceed 3 min per animal. After stabilization, animals were exposed to 2 h hypoxia (8% O_2_, 92% N_2_) in an airtight container maintained at 37°C. Rat pups were then returned to their dams.

In this model, humane endpoints were used. Humane endpoints were composed of body weight growth (1: slower growth than normal rats, 2: growth arrest, 3: weight loss), responsiveness (1: delayed but appropriate response, 2: delayed and null response, 3: no response), and appearance (1: rough hair coat, 2: porphyrin staining, 3: sustained abnormal posture or dilated pupil). We assessed and monitored the condition of animals on a weekly basis regularly and four times per day in a daily basis especially for the seven days after modeling. Based on the above scoring system, total scores over than 5 or scores over than 3 in a single category were determined as humane endpoint. To minimize the suffering of the rats, we fed the rats those showed slower weight gain than normal rats, with supplementary artificial milk at least more than four times per day. Euthanasia was planned to be performed via a deep pentobarbital (Entobar ®, Hanlim Pharmaceutical C7-8o., Seoul, Korea) anesthesia (60 mg/kg, intraperitoneal), however, no rats in the present study met the humane endpoint before sacrifice.

*In vivo MRI Assessment*

Brain MRI was used to confirm severe baseline brain injury after HIE modeling and to monitor changes in damage at P12 and P42. MRI was performed using a 7.0-Tesla MRI system (Bruker-Biospin, Fällanden, Switzerland) as described previously[[6](#_ENREF_6),[7](#_ENREF_7)]. Lesions were identified as hyperintense areas in DWI performed 2 h after HI and hyperintense areas in T2-weighted imaging at P12 and P42. The intact ipsilateral-to-whole-contralateral hemispheric brain volume ratio was calculated as previously reported as a measure of brain injury[[7](#_ENREF_7)]. Volume estimates were according to Cavalieri's principle[[6](#_ENREF_6),[7](#_ENREF_7)]. The investigator was blinded to the treatment group.

*Transplantation of Donor Cells with Stereotaxis*

For donor cell transplantation, 1 × 10^5^ human UCB-derived MSCs in 10 μl saline were administered into the ipsilateral right lateral ventricle using a stereotactic method[[6](#_ENREF_6),[7](#_ENREF_7)] (Digital Stereotaxic Instrument with fine drive, MyNeurolab, St. Louis, MO, USA; coordinates, x = +0.5, y = +1.2, z = -2.7 mm relative to bregma) 6 h after HI. For HNC and HHC groups, an equivalent volume of saline was administered using the same method.

*Post-Ischemic Temperature Modulation*

For the temperature intervention, pups were separated from mothers and placed in a temperature-controlled chamber for 24 h, separated by plastic containers. Ambient temperature inside the chamber was 34.0°C for normothermia (HNC, MNC) and 31.0°C for hypothermia (HHC, HHM). During the temperature intervention, rectal temperature was measured every 2 hours with a flexible temperature probe, and temperatures were maintained at 35.5–36.5°C for normothermia and 31–32°C for hypothermia (Supplementary Fig. 1). Throughout the temperature intervention, pups were fed five times per day with 0.5 ml of milk formula using a 22-G animal feeding needle. Pups were returned to dams until imaging and behavioral studies at P42.

*Donor Cell Tracking*

Localization of transplanted donor cells labeled with micron-sized paramagnetic iron-oxide (MPIO) particles (Bangs Laboratories, Fishers, IN, USA) was observed by T2*-weighted brain MRI at P42. After the rats had been sacrificed, 10-µm-thick cryosections were cut from brains at the medial septum area (+0.95 mm to -0.11/bregma) and mounted with Vectorshield mounting solution containing DAPI (H-1200, Vector Laboratories, Inc., Burlingame, CA, USA). Localization of MPIO green fluorescent cells in coronal sections was assessed by confocal microscopy (Biorad Radiance 2100, Bio-Rad Laboratories, Inc., Hercules, CA, USA).

*TUNEL Assay*

Immunofluorescent terminal deoxynycleotidyltransferase-mediated deoxyuridine triphosphate nick end-labeling (TUNEL) (kit S7110 ApopTag, Chemicon, Temecula, CA, USA) was used to evaluate apoptosis in brain sections according to the manufacturer’s protocol as previously described[[6](#_ENREF_6),[7](#_ENREF_7)]. Three coronal sections (+0.95 mm to -0.11 mm/bregma) were chosen from each brain, and three random nonoverlapping fields were selected in the cortical penumbra region from each section. The number of TUNEL-positive nuclei in selected fields was counted by an evaluator who was blind to the experimental groups.

*Immunohistochemistry*

Immunofluorescence histochemical staining was performed for GFAP (rabbit polyclonal; Dako, Glostrup, Denmark) as an astrocytic glial marker and ED-1 (mouse monoclonal; Millipore, Concord Road, MA) as a marker for reactive microglia as previously reported[[6](#_ENREF_6),[7](#_ENREF_7)]. Three coronal sections (+0.95 mm to -0.11 mm/Bregma) were stained from each section, and three random nonoverlapping fields in the cortical penumbra area were selected from each section. The optical density of immunofluorescence in fields was measured using Image J software by an observer blind to the experimental groups (National Institutes of Health, USA).

*Enzyme-linked Immunosorbent Assay*

Frozen CSF samples at -70°C were thawed and centrifuged at 2,000 × *g* for ten minutes at 4°C. Interleukin (IL)-1α, IL-1β, IL-6, and tumor necrosis factor (TNF)-α concentrations in CSF were measured using Milliplex MAP enzyme-linked immunosorbent assay (ELISA) kits according to the manufacturer’s protocol (Millipore, Billerica, MA, USA)[[6](#_ENREF_6)].

*Behavioral Tests*

To assess sensorimotor function, rotarod tests were performed at P40 and 41 by analyzing latency to fall[7,8]. All animals were tested three times on two consecutive days with a 15-min intertrial interval. As rats can learn rotarod tests, the values were analyzed by date, and the average latency to fall from the three trials was used.

At P42, cylinder tests were performed by analyzing forelimb movement within a transparent cylinder (25 cm diameter and 40 cm height) as previously described[[7](#_ENREF_7)]. Briefly, the first limb to contact the wall was scored as an independent wall placement for that limb. If an animal placed both paws on the wall, both limbs were scored. Limb use asymmetry was calculated using the following formula: 100 × number of (left forelimb contacts)/(right forelimb contacts + left forelimb contacts). Each animal was video recorded for 5 min per session. All measurements were recorded three times per animal. The evaluator was blind to the treatment groups.

Supplementary references

1. Chang YS, Oh W, Choi SJ, Sung DK, Kim SY, et al. (2009) Human umbilical cord blood-derived mesenchymal stem cells attenuate hyperoxia-induced lung injury in neonatal rats. Cell Transplant 18: 869-886.

2. Boiani M, Scholer HR (2005) Regulatory networks in embryo-derived pluripotent stem cells. Nat Rev Mol Cell Biol 6: 872-884.

3. Gang EJ, Bosnakovski D, Figueiredo CA, Visser JW, Perlingeiro RC (2007) SSEA-4 identifies mesenchymal stem cells from bone marrow. Blood 109: 1743-1751.

4. Jang YK, Jung DH, Jung MH, Kim DH, Yoo KH, et al. (2006) Mesenchymal stem cells feeder layer from human umbilical cord blood for ex vivo expanded growth and proliferation of hematopoietic progenitor cells. Ann Hematol 85: 212-225.

5. Yang SE, Ha CW, Jung M, Jin HJ, Lee M, et al. (2004) Mesenchymal stem/progenitor cells developed in cultures from UC blood. Cytotherapy 6: 476-486.

6. Ahn SY, Chang YS, Sung DK, Sung SI, Yoo HS, et al. (2013) Mesenchymal stem cells prevent hydrocephalus after severe intraventricular hemorrhage. Stroke 44: 497-504.

7. Kim ES, Ahn SY, Im GH, Sung DK, Park YR, et al. (2012) Human umbilical cord blood-derived mesenchymal stem cell transplantation attenuates severe brain injury by permanent middle cerebral artery occlusion in newborn rats. Pediatr Res 72: 277-284.
